# The modulation of ion channels in cancer chemo-resistance

**DOI:** 10.3389/fonc.2022.945896

**Published:** 2022-08-10

**Authors:** Jiayu Zhao, Mei Li, Jiao Xu, Wei Cheng

**Affiliations:** Institute of Cancer Stem Cell, Dalian Medical University, Dalian, China

**Keywords:** ion channel, drug resistance, tumor microenvironment, cancer stem cell, metabolism

## Abstract

Ion channels modulate the flow of ions into and out of a cell or intracellular organelle, leading to generation of electrical or chemical signals and regulating ion homeostasis. The abundance of ion channels in the plasma and intracellular membranes are subject to physiological and pathological regulations. Abnormal and dysregulated expressions of many ion channels are found to be linked to cancer and cancer chemo-resistance. Here, we will summarize ion channels distribution in multiple tumors. And the involvement of ion channels in cancer chemo-resistance will be highlighted.

## Introduction

Drug resistance may be intrinsic (i.e., present prior to chemo-therapy), and tumor cells are prone to rapidly emerge acquired resistant to conventional therapies. Indeed, although systemic agents (cytotoxic, hormonal, and immune-therapeutic agents) used for cancer treatment are usually effective at the very start (e.g., 90% of primary breast cancers and 50% of metastatic cancers), approximately 30% of breast cancer patients in early-stage would have recurrence due to acquired resistance (1). Study indicated that tumor cells have increased resistance to chemo-therapeutic agents in recurrent tumors (2). And continuous exposures to chemo-therapeutic agents then promote the development of acquired resistance in these cells, leading to subsequent failure of chemo-therapy (3). Moreover, residual tumor cells which remain quiescent before resuming still can be detected in most patients after treatment. And then result in tumor recurrence.

Ion channels are the basic excitatory units on the membranes of many tissue cells, such as nerves, muscles, and glands, which can generate and conduct electrical signals and have important physiological functions. Ion channels are not only directly associated with excitability, but also can further influence and control transmitter release, gland secretion, and maintenance of constant cell volume and internal environmental stability. Gating of ion channels and the ensuing ion fluctuation is a highly complex process that involves in ion homeostasis, the initiation of signaling networks, and changes in microenvironment. Increasing studies demonstrated that ion channels not only expressed in excitable nerve cells and tissues, but also distributed in cancer cells and tissues. Altered ion channel expression is considered as a hallmark of several types of cancer, and some ion channels have been linked with chemo-resistance in cancers (4, 5). In the present review, we will summarize the involvement of ion channels in multiple tumors’ chemo-resistance, with emphasis on their molecular mechanism (6). Deciphering the mechanisms of ion channels involving in mediating multiple tumors chemo-resistance may provide new avenues for targeted cancer treatment.

## Ion channels in cancer: Expressions and their implications

Ion channels were initially divided into two main classes of voltage-gated channels (VGC) and ligand-gated channels (LGC). Different VGC channel types are distinguished by the ions (calcium, potassium, sodium, chloride) through which they most selectively pass. While LGC channel types are distinguished according to the signaling molecules (ligand) which specifically activate them (e.g., GABA, acetylcholine, glutamate, glycine, 5-hydroxytryptamine). Since LGCs act as receptors for those signaling molecules, they are often referred to as their respective receptors as well (7). With the development of gene cloning technology and the understanding of different gating mechanisms, ion channels have been linked to physical (light, temperature, pressure, tension) and chemical (pH, PO_2_, contaminants, cooking spices) stimuli, as well as intracellular factors such as ATP levels, organelle status, presence of second messengers, thus they may involve in physiological and pathological functions.

### Calcium-permeable ion channels

Ca^2+^-permeable channels are classified by their intracellular residence of either on plasma membrane (PM) or in ER (endoplasmic reticulum) membrane. PM Ca^2+^-permeable channels include six major subclasses which distinguished by their principal activation mechanisms: 1) VGCC for “voltage-gated calcium channels, Cav” can be further divided into three subfamilies of Cav1 or L-type (Cav1.1, Cav1.2,Cav1.3, Cav1.4); Cav2 (Cav2.1 or P/Q-type, Cav2.2 or N-type, Cav2.3 or R-type); and Cav3 or T-type (Cav3.1, Cav3.2, Cav3.3) (8); 2) LGC for “ligand-gated channels” (9); 3) SOC for “store-operated channels” (10); 4) TRP for “transient receptor potential” channels (11); 5) SMOC for “second messenger-operated channels” can be divided into two groups of cyclic nucleotide-gated channels (CNGA1-4, CNGB1, CNGB3) and arachidonate-regulated Ca^2+^ channels (IARC) (12–14); and 6) Mechano-gated channels (15).

The Cav channels are primarily responsible for the entry of Ca^2+^ into excitable cells such as neurons as well as neuron-like cells and various types of muscle cells. They open during membrane depolarization and Ca^2+^ influx through them, allowing electrical excitation combined with activation of specific cellular responses. The Cav channels have been found involved in the development of various types of cancer, including brain cancer, colorectal cancer, gastric cancer, pancreas cancer, breast cancer, prostate cancer, bladder cancer, lung cancer, esophagus cancer, ovarian cancer, cervix cancer, renal cancer, leukemia, neuroblastoma, glioblastoma, and sarcoma, etc. In breast cancer and leukemia, the Cav channels exhibited up-regulated (16, 17). The T-type calcium channels of Cav3.2 have been observed present in human prostate cancer cells. During neuroendocrine differentiation, Cav3.2 channels are up-regulated with increasing basal calcium entry. It suggests that the Cav3.2 may serve in facilitating prostate cancer development (18). Similarly, Cav3.1 are found over-expressed in prostate cancer. Knockdown of Cav3.1 inhibits the cell proliferation, migration and invasion by suppressing AKT activity in prostate cancer cells (19).

Less diverse Ca^2+^-permeable channels on the ER membrane are classified according to Ca^2+^-mobilization mechanism: 1) Ca^2+^-induced Ca^2+^ release (CICR) and 2) agonist-induced GPCR-dependent Ca^2+^ release. CICR is mediated through Ca^2+^ release channels on the ER membrane, termed ryanodine receptors (RyR). The RyR is a homo-tetramer assembly of subunits for homologous genes encoded with RyR1 (primarily skeletal muscle), RyR2 (primarily cardiac), and RyR3 (ubiquitous) (20). Its primary physiological ligand is intracellular Ca^2+^ per se (which is the initiation of the name CICR). Moreover, CICR also can be activated both *via* interaction with some members of Cav family and by cytoplasmic cyclic ADP-ribose (cADPR). Inositol trisphosphate receptor (IP3R) is an agonist-induced, GPCR-dependent Ca^2+^ release channel. Calcium permeable channels are key players in mediating numerous physiological and pathological functions. Intracellular Ca^2+^ homeostasis affected cell cycle, apoptosis, autophagy, migration. Further, it also involved in regulation of release of neurotransmitters, hormones and growth factors in both normal and neoplastic cells (21–23).

### Potassium ion channels

K^+^ channels comprise voltage-gated K^+^ channels (Kv), calcium-activated K^+^ channels (KCa), inward-rectifier potassium channels (Kir, IRK), and two-pore domain K^+^ channels (K2p) (24).

Given its high distribution and functional relevance in tumor tissues, Kv11.1 (hERG) channel which belongs to the voltage-gated Kv family has been deemed to potential anticancer target (25). Arcangeli and co-workers found that hERG channels promoted proliferation in neuroblastoma cells *via* controlling membrane resting-potential (26). Study revealed that over-expression of voltage-gated Kv channel of Kv10.1 (EAG1) enhanced cell proliferation and conferred a transformed phonotype with oncogenic potential (27). Further, EAG1 channels have been detected in approximately 70% of human tumor biopsies originated from osteosarcoma, pituitary adenomas, glioblastoma, head and neck cancer, ovarian cancer, leukemia, gastric cancer and colorectal cancer (28). To date, pharmacological targeting EAG1, hERG for the treatment of cancer have drawn much attention. A monoclonal antibody specifically against EAG1 has been identified to suppress colony formation of several cancer cell lines and tumor growth *in vivo via* inhibiting channel function (29). Over-expression of hERG in cancer cells involved in regulating of tumor progression and migration *via* co-assembly with β1 integrin related adhesion-dependent signaling complex (30, 31). Thus, both *in vitro* and *in vivo* models illustrated very convincingly that EAG1 as well as hERG can be act as promising oncological targets. Studies targeting other K^+^ channels also point to an important role of K^+^ channels in tumor progression. KCNQ1 encodes the pore-forming α subunit of voltage-gated potassium channels and they are considered to be a tumor suppressor in colorectal cancer. Inhibition of the KCNQ1 channels lead to colorectal cancer cell proliferation, EMT and tumorigenesis (32). KCNQ1 channels also act as a tumor suppressors in gastrointestinal and esophageal cancers (33, 34). Using human A549 lung adenocarcinoma model, researchers found that either blockade or suppression of Kv1.3 could significantly inhibit cell proliferation and reduced tumor volume by 75% *in vivo* (35). In addition, calcium-dependent potassium channels and the two pore TASK-3 channels have been demonstrated possessing oncological potentials (36, 37).

### Sodium ion channels

Na^+^ channels include VGSC (voltage-gated sodium channel) and LGSC (ligand-gated sodium channel) subfamilies. VGSC comprises nine subtypes of Nav1.1 ~ Nav1.9 containing both α and β subunits.

The expression of VGSC, particularly for Nav1.5, Nav1.6 and Nav1.7 and their splicing variants were found up-regulated in many cancer types, including prostate, breast, lung, cervical cancer, and leukemia (38). In breast cancer cells, Nav1.5, Nav1.6 and Nav1.7 are all present. In particular, a novel neonatal isoform of Nav1.5 (nNav1.5) exhibited up-regulation during breast cancer progression. And the channel activity of Nav1.5 enhances cellular metastatic cascade both *in vitro* and *in vivo* (39). In addition, another study specified that Nav1.6 channels expressed in cervical cancer cells and tissues. During cancer development, Nav1.6 was significantly up-regulated with channels activities and then induced the secretion of matrix metalloproteinase type 2 (MMP-2), promoting cancer cells invasion and metastasis (40). Moreover, Nav1.5 channels activities could enhance aggressiveness by stimulating cysteine cathepsin in breast and NSCLC cancer cells (41).

### Transient receptor potential ion channels

Currently, transient receptor potential (TRP) ion channel proteins are emerging as promising oncological targets (42, 43). TRP ion channels can be divided into six subfamilies, namely TRPV (vanilloid), TRPA (ankyrin), TRPM (melastatin), TRPC (canonical), TRPML (mucolipin) and TRPP (polycystin). Mammalian TRP subunits can be formed by homo- or hetero- tetramerization of non-selective cation channels that can be stimulated by a variety of different factors, including temperature changes, mechanical stress, osmotic pressure, changes of O_2_ and pH, ROS, growth factors and cytokines. Therefore, they are expected to play critical roles in tumor microenvironment crosstalk. TRPC channels are activated through pathways coupled to phospholipase C (PLC), and can support receptor-operated Ca^2+^ entry; TRPC1 and TRPC4 can also contribute to store-operated Ca^2+^ entry (SOCE) *via* relatively non-selective cationic currents.

Several investigations found evidence that TRPC ion channels function in the regulation of cancer process (44). A study reported that TRPC1 channels expressed in human glioma cells as well as glioblastoma biopsies. Knock-down of TRPC1 clearly suppressed cell proliferation and decreased tumor volume by 40% in a xenograft model using human grade IV glioma D54MG cells (45). TRPC4 channels expression lost in the cells of renal carcinoma. The absence of TRPC4 may cause decreased calcium uptake and then enrich an angiogenesis inhibitor of the secreted TSP1(thrombopsondin-1) in the cytoplasm which subsequently suppress angiogenesis during renal cell carcinoma progression (46). Among TRPVs, the highly calcium-selective channel of TRPV6 which allows the passage of heavy metals zinc, manganese and cadmium (47) has been found expressed in prostate and breast cancers. Its expression correlates with cancer progression, suggesting that it drives cancer cell growth. TRPV2 was over-expressed at both mRNA and protein levels in esophageal squamous cell carcinoma (ESCC) cell lines. Knockdown of TRPV2 gene decreased cell proliferation, cell cycle progression and migration (48). *In vitro* and *in vivo*, high levels of TRPV4 expression were associated with tumor metastasis. Proteomics and bioinformatics analyses have shown that TRPV4 was involved in regulating the cytoskeleton and Rho protein pathway of cell migration in endometrial cancer (49).

### Chloride ion channels

Chloride ion channels can be roughly classified into voltage-gated chloride channel (ClC), ligand-gated chloride channel, calcium-activated chloride channel (CaCC), High conductance chloride channels, cystic fibrosis transmembrane conductance regulator (CFTR), volume-regulated chloride channels (VRCC), and chloride intracellular channel (CLIC) (50). A study showed that ClC-3 anion channels promoted brain tumor metastasis (51). Chlorotoxin purified from Leiurus scorpion, a chloride channel inhibitor, has been identified to suppress glioma cell invasion *via* binding to MMP-2, and voltage-gated chloride channel were specifically expressed in human astrocytoma and glioma cells (52, 53). This chloride channel was subsequently identified as ClC-3, a type of Cl^-^/H^+^ exchanger mainly expressed in endosomal/lysosomal compartments (>95%). And chlorotoxin may inhibit cell migration and invasion by interacting with both Cl- channel proteins and MMP-2 in glioma cells (54, 55).

ANO1/TMEM16A, a member of the CaCC functioned in maintaining ion and tissue homeostasis *via* regulating epithelial secretion and cell volume (56). ANO1/TMEM16A is highly expressed in several epithelium originated carcinomas, gastrointestinal stromal tumor, esophageal squamous cell carcinoma (ESCC) and pancreatic cancer. Knockdown of ANO1 inhibited cell proliferation, induced cell apoptosis in breast and lung cancer cells, and reduced tumor growth in established cancer xenografts (57–59). While decreasing ANO1 confers metastatic phonotype in squamous cell carcinoma of head and neck. Stable reduction of ANO1 expression enhanced cell motility and metastases, but decreased tumor proliferation in an orthotopic mouse model (60). Thus, suppression of chloride channel may be a hopeful target for clinic practice by small molecule screening as well as *in vivo* studies (61, 62).

## Ion channels modulate chemo-resistance through tumor microenvironment

Cancer progression and metastasis depend on bidirectional interactions between cancer cells and their environment, which together form tumor microenvironment (TME) (63). The TME is a complex, dynamic network composed of cellular and non-cellular components (64, 65). And the TME has been characterized by hypoxia, an acidic extracellular pH, high lactate levels, elevated adenosine concentrations, low levels of glucose, ATP and nutrients, and the presence of vascular endothelial growth factor (VEGF) and other cytokines and growth factors (66–68). Among these factors, hypoxia is of particular concern. Solid tumors generally contained hypoxic regions that can trigger important cellular changes (69). Moreover, cancer cells metabolize glucose in the form of glycolysis (‘Warburg effect’), and hypoxia can further aggravate the dependence on glycolytic fueling, which resulted in the production of large amounts of lactic acid (70, 71). Although resistance is a characteristic of cancer cells evolving in a low-oxygen environment (hypoxia), the mechanisms involved remain elusive (72).

### Calcium-permeable ion channels

Mibefradil is an orally bio-available T and L-type calcium channel blocker for the treatment of hypertension. The expression of T-type calcium channel of Cav3.2 was increased in glioblastoma (GBM) cells and glioblastoma stem-like cells (GSCs). Mibefradil suppresses Cav3.2 ion channels activity, and then subdues pro-survival AKT/mTOR pathways and up-regulate phosphorylation of LKB1 and Tuberin/TSC2, thus inhibiting cell proliferation. Meanwhile, inhibition of Cav3.2 by mibefradil could activate BAX, caspase-9 and PARP signalings, enhancing GSCs apoptosis (73).

It is well known that hypoxia can induce stem cell-like transcriptional program *via* HIFs (hypoxia-inducible factor), as described for breast cancer stem cells, prostate and glioma stem like cells, even human embryonic stem cells (74, 75). Under hypoxic conditions, Cav3.2 expression was up-regulated in GSCs with high level of HIFs. Notably, application of calcium channel blocker of mibefradil could down-regulate the expression of HIF-1α and HIF-2. It is suggested that mibefradil may suppress GSC malignant parameters by reducing hypoxic pressure and inhibiting expression of HIFs (73).

### Potassium ion channels

Recent studies have shown that large-conductance, calcium-activated potassium (BK) channels promoted several aspects of the aggressive potential induced by hypoxia, such as migration and chemo-resistance to cisplatin in glioblastoma (GBM) cells, suggesting it may act as a potential therapeutic target in GBM (76).

BK channels are expressed in GBM cells and channel activity could affect tumor aspects, such as migration/invasion, and cell death. GBMs are also characterized by a heavy hypoxic microenvironment that exacerbates tumor aggressiveness. In human GBM U87 MG cells, hypoxia promoted cell migration as well as spheroids formation, and induced chemo-resistance to cisplatin. And inhibition of BK channels with paxilline could diminish cells migration and chemo-resistance to cisplatin induced by hypoxia. Moreover, BK channels were also found to be essential for hypoxia-induced differentiation of GBM cells (76).

### Transient receptor potential ion channels

Recent study indicates that over-expression of TRPC6 regulated multi-drug resistance (MDR) by elevation of intracellular calcium under hypoxia, or stimuli of doxorubicin and ionizing radiation in hepatocellular carcinoma. In response to these stimuli, intracellular calcium ions accumulation persisted, and inhibition of calcium signaling pathways enhanced cellular sensitivity to various drugs by inhibiting epithelial-mesenchymal transition (EMT), HIF-1α signaling pathway and DNA repair. Specifically, the use of siRNA to down-regulate the expression of Twist, HIF-1α and H2A.X significantly attenuated MDR. Moreover, blockade of TRPC6 by either siRNA or SKF-96365 can diminish MDR induced by various stimuli *in vitro*. An *in vivo* xenograft model of liver cancer further confirmed that inhibition of TRPC6 enhanced the efficacy of doxorubicin. These results suggested that the regulatory mechanisms of MDR in hepatocellular carcinoma cells were calcium-dependent *via* the TRPC6/calcium/STAT3 pathway (77). In addition, TRPC6 was also involved in regulating tumor malignancy. Under hypoxia, TRPC6 expression increased with a sustained elevation of intracellular calcium *via* agonism in glioblastoma U373 MG cells. And it was required for the development of malignant phenotype of GBM (78). In hypoxic microenvironment, tumor cells are mainly regulated by hypoxia-induced transcription factor HIF-1. A study has showed that TRPM8 over-expressed in advanced prostate cancer, and TRPM8 promoted cancer cell growth *in vitro* hypoxia, drug resistance, and *in vivo* tumorigenicity, with increased HIF-1α expression. These effects were further enhanced by activation of TRPM8 but inhibited by suppression of TRPM8 (79).

Another study showed that hypoxia can simultaneously increase the expression of TRPM7 and induce the accumulation of HIF-1α in androgen-independent prostate cancer cells. Silencing TRPM7, however, significantly promoted the degradation of HIF-1α and inhibited EMT changes in hypoxic conditions (80). Recent studies have demonstrated that HIF-1α promoted the proliferation, migration, invasion, angiogenesis and EMT in gastric cancer (GC) cells (81). In addition, hypoxia can induce autophagy and an acidic extracellular pH which correlated with GC progression and chemo-resistance (82–85).

### Other ion channels

**Figure 1:**
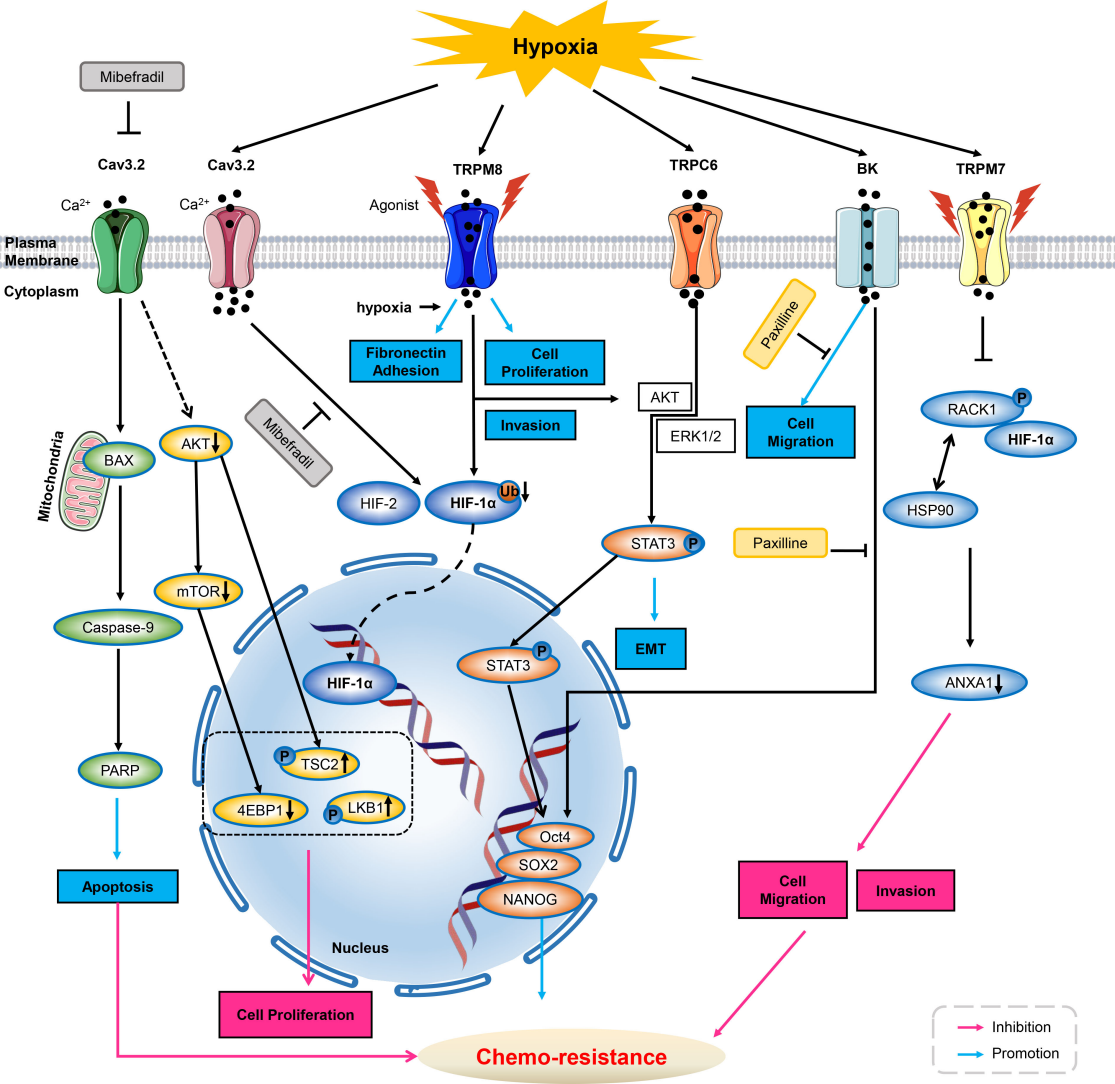
Ion channels modulate chemo-resistance through tumor microenvironment. As inhibitor of T-type calcium ion channels, mibefradil suppresses Cav3.2 ion channel activity, and then decreases AKT, mTOR and 4EBP1 activities and up-regulated phosphorylation of LKB1 and Tuberin/TSC2, thus inhibiting cell proliferation. Meanwhile, inhibiting Cav3.2 by mibefradil activates BAX, caspase-9, PARP, thus enhancing GSCs apoptosis. Cav3.2 can be up-regulated by hypoxia, and application of Cav3.2 inhibitor of mibefradil inhibits HIF-1α and HIF-2 in GSCs. In hypoxia, menthol or icilin stimulation promotes cell proliferation and invasion. Simultaneous hypoxia stimulation increases fibronectin adhesion and further enhances menthol treatment. TRPM8 over-expression enhances HIF-1α in hypoxia-exposed prostate cancer cells by reduction of HIF-1α ubiquitination. Hypoxia increases the expression of TRPC6, then activates AKT/ERK1/2 to promote phosphorylation of STAT3, inducing its nucleus translocation, triggers the expression of Oct4, SOX2 and NANOG, thus deteriorating chemo-resistance. HIF-1α increases upon BK channel blocker of Paxilline in GBMs. Hypoxia increased expression and the nuclear translocation of stemness markers of Oct4, NANOG and SOX2, therefore increasing chemo-resistance to Paxilline in GBMs. Hypoxia increases TRPM7 expression and enriches HIF-1α in prostate cancer cells. Suppression of TPRM7 enhances phosphorylation of RACK1 and promotes the binding of RACK1 to HIF-1α by competing with HSP90, then inhibiting downstream signaling of ANXA1 to suppress the migration and invasion, thus weakening chemo-resistance in prostate cancer cells. GSC, glioblastoma stem-like cells; HIF, hypoxia inducible factors; AKT, protein kinase B; mTOR, mammalian target of rapamycin; BK, calcium-activated potassium channels; GBM, glioblastoma; PARP, poly ADP-ribose polymerase; ANXA1, Annexin A1; ERK1/2, extracellular regulated protein kinases1/2; STAT3, signal transducer and activator of transcription 3.

The truncated voltage-dependent anion channel 1 (VDAC1-ΔC), can be found in certain hypoxic cells and were linked to chemo-resistance *via* interaction with Bcl-xL and hexokinase I. The formation of truncated VDAC1 was dependent on HIF-1 and can be inhibited in the presence of the tetracycline antibiotics doxycycline and minocycline (known metalloproteinase inhibitors). Interestingly, VDAC1-ΔC has been detected in lung adenocarcinoma tumor tissue of patients. Therefore, targeting VDAC1-ΔC may provide a strategy for combating chemo-resistance (72) (**Figure 1**).

## Ion channels modulate chemo-resistance through cancer stem cells

Cancer stem cells (CSCs) have been identified in many cancer types (86–94). CSC was a small fraction of the cells that yet remain in the patient after conventional antitumor therapy completed (95). CSCs have also been described to be responsible for tumorigenesis as well as stemness maintenance with characteristics of self-renewal ability, asymmetric cell division, slow division kinetics, invasion, metastasis, enhanced tumor formation and proliferation, resisting apoptosis and resistance to conventional chemo-therapy and radio-therapy (96–100). CSCs can be recognized by a variety of cellular markers (87, 101–103). Cell surface markers such as CD133, CD44, CD87 and ALDH1 are commonly used to isolate and enrich the CSC populations. Three essential transcription factors of Oct4, NANOG, and SOX2 expressed in both tumor stem cell-like cells and embryonic stem cells are described as stem cell markers. CSCs are thought to evade conventional treatment and are responsible for chemo-resistance and recurrence of cancer.

Side-population (SP) cells facilitate the extrusion of exogenous compounds for detoxification of cells by expressing ATP transporter proteins. The SP cells are clearly enriched in stem cells, and the SP phenotype may account for the chemo-resistance of a subpopulation of tumor cells (104–106). Recent studies on the SP cells have confirmed that this particular group of cells not only contributed to the resistance of tumor cells to chemo-therapeutic drugs, but also were closely associated with proliferation, differentiation and stemness of cancer cells (107, 108).

### Calcium-permeable ion channels

Calcineurin mediating the dephosphorylation and activation of nuclear factor of activated T-cells (NFAT), originally was associated with promoting T-cell proliferation but more recently linked to proliferation, migration and resistance across various cancer types (109). Studies indicated that calcium influx through TRP channels as well as other calcium channels modulated the activations of NFAT and ERK pathways in cancer cells (43, 110, 111). T-type VGCC of Cav3.2 has been observed up-regulated in the glioblastoma stem-like cells and blockade of these channels with mibefradil suppressed both growth and stemness of GSCs (73). Up-regulated Cav3.2 expression in GBM was associated with poor prognosis, suggesting that Cav3.2 has the potential for treatment of GBM and may improve patient survival. Mibefradil sensitized GSCs to temozolomide, a key chemo-therapeutic agent used for GBM treatment. GSCs have been partially mediating resistance both to chemo-therapy and radio-therapy. Notably, resistant GSCs survived and maintained malignant growth of GBM after surgical intervention and chemo-therapy (112–114). Studies have shown that mibefradil induced differentiation of GSCs, as evidenced by down-regulation of stemness markers for CD133, Nestin, Bmi1 and SOX2, and up-regulation of the differentiation markers for GFAP, Tuj1 and MAP2. Although Cav3.2 inhibition strongly impaired the malignant parameters of GSC, it may also affect differentiated bulk GBM cells, as shown in U87 MG cell line (115).

Drug resistance in epithelial ovarian cancer has been attributed to the persistence of tumor stem cells. A small number of drug-resistant CSCs survived from chemo-therapy, leading to recurrence and aggressive proliferation of ovarian cancer (116). Studies have shown that the long-term efficacy of chemo-therapy depends on the prevention of recurrence *via* targeting CSCs (117, 118). Lee and co-workers identified four voltage-gated calcium channel blockers (manidipine, lacidipine, benidipine, and lomerizine) that targeted ovarian stem cells *via* screening a library of FDA-approved compounds. The four calcium channel blockers (CCBs) reduced sphere formation, cell proliferation, and induced apoptosis in ovarian stem cells. The CCBs disrupted the stemness *via* inhibiting AKT and ERK signaling pathways in ovarian cancer stem cells. Study indicated that three of L- and T-type calcium channels were over-expressed in ovarian CSCs, and down-regulation of calcium channels reduced the stem cell-like properties of ovarian cancer cells. Further, expressions of these calcium channels were negatively correlated with the survival rate of the patients. Treatment using CCBs in combination with cisplatin could effectively inhibit the proliferation of CSCs, suggesting that the combination therapy could improve the drug sensitivity of the CSC-enriched epithelial ovarian cancer population. In addition, combined with manidipine and paclitaxel showed enhanced effects in a mouse model of ovarian CSCs xenografts. The results suggested that the four CCBs may be potential therapeutic agents for the prevention of ovarian cancer recurrence (119).

Studies have identified a composing subunit of a voltage-dependent calcium channel of α2δ1 as a promising marker for CSCs. Recent insight from Zhao and co-workers has reported that α2δ1^+^ cells presented in primary hepatocellular carcinoma (HCC) cell with CSCs properties. They have found that α2δ1 was a functional marker for predicting HCC recurrence and its monoclonal antibody 1B50-1 can be used as a potential anti-HCC drug (120). Another study has identified that α2δ1 positive cells possessed tumor stem cell properties that may be associated with chemo-resistance in small cell lung cancer cells. Also, the use of 1B50-1 antibody in patient-derived xenograft models could help overcome chemo-resistance and delay the recurrence of small cell lung cancer (121). Among them, stem cell-related transcription factors of SOX2, Oct4, NANOG and drug resistance-related genes (such as MDR, ABCG2) were highly expressed in α2δ1^+^ and CD133^+^ cells, especially in α2δ1^+^ cells. In addition, α2δ1^+^/CD133^+^ cells exhibited higher sphere-forming ability and differentiation characteristics than CD133^+^ cells *in vitro*. Meanwhile, α2δ1^+^ cells showed higher growth rate and proliferation ability than CD133^+^ cells. Moreover, α2δ1 over-expression may be associated with chemo-therapy resistance both in small cell lung cancer as well as in liver cancer (120, 121). And activation of MAPK pathway may mediate drug resistance (122).

### Potassium ion channels

Recent findings suggested that KCa1.1 in LNCaP spheroids, which mimic human prostate cancer (PC) stem cells, has the potential to be a therapeutic target for overcoming anti-androgen and doxorubicin resistance in PC cells (123). While another study has evaluated the targeting effect of trimebutine maleate (TM) on ovarian CSCs. TM is used as a modulator for gastrointestinal motility, and it is a agonist of peripheral opioid receptor and a blocker of multiple channels. Voltage-gated calcium channels (VGCC) and calcium-activated potassium channels (KCa) were over-expressed on ovarian CSCs and targeted by TM, inhibition of both channels reduced the survival of ovarian A2780-SP cells. TM reduced the expression of stemness-associated proteins; simultaneous inhibition of VGCC and KCa could reproduce this trend compared to single-channel inhibition. In addition, TM inhibited the Wnt/β-catenin, Notch and Hedgehog pathways, which were implicated in many features of CSCs. Inhibition of these ion channels by TM could active β-catenin signaling *via* ERK1/2 phosphorylation and reduce the expression of transcriptional factors of Oct3/4 and SOX2, inhibit cell growth of ovarian CSCs, therefore abating chemo-resistance (124).

### Transient receptor potential ion channels

More recently, TRPV2 has been directly linked to self-renewal of CSCs in a number of cancer types such as esophagus cancer (125), liver cancer (126) and glioblastoma (127). Glioma stem cells (GSCs) correspond to a subpopulation of tumor cells involved in tumor initiation and acquired chemo-resistance in glioblastoma multiforme (GBM). Currently, drug-induced differentiation is considered as a promising approach to eradicate this tumor-driven cell population. Studies have demonstrated that cannabidiol (CBD) activated the process of autophagy by triggering the differentiation of GSCs through the activation of TRPV2. The acute myeloid leukemia (AML) -1a was up-regulated during differentiation of GSCs. CBD up-regulated AML-1a expression in a TRPV2 and PI3K/AKT-dependent manner and eliminated chemo-resistance of GSCs to Carmustine (127).

**Figure 2:**
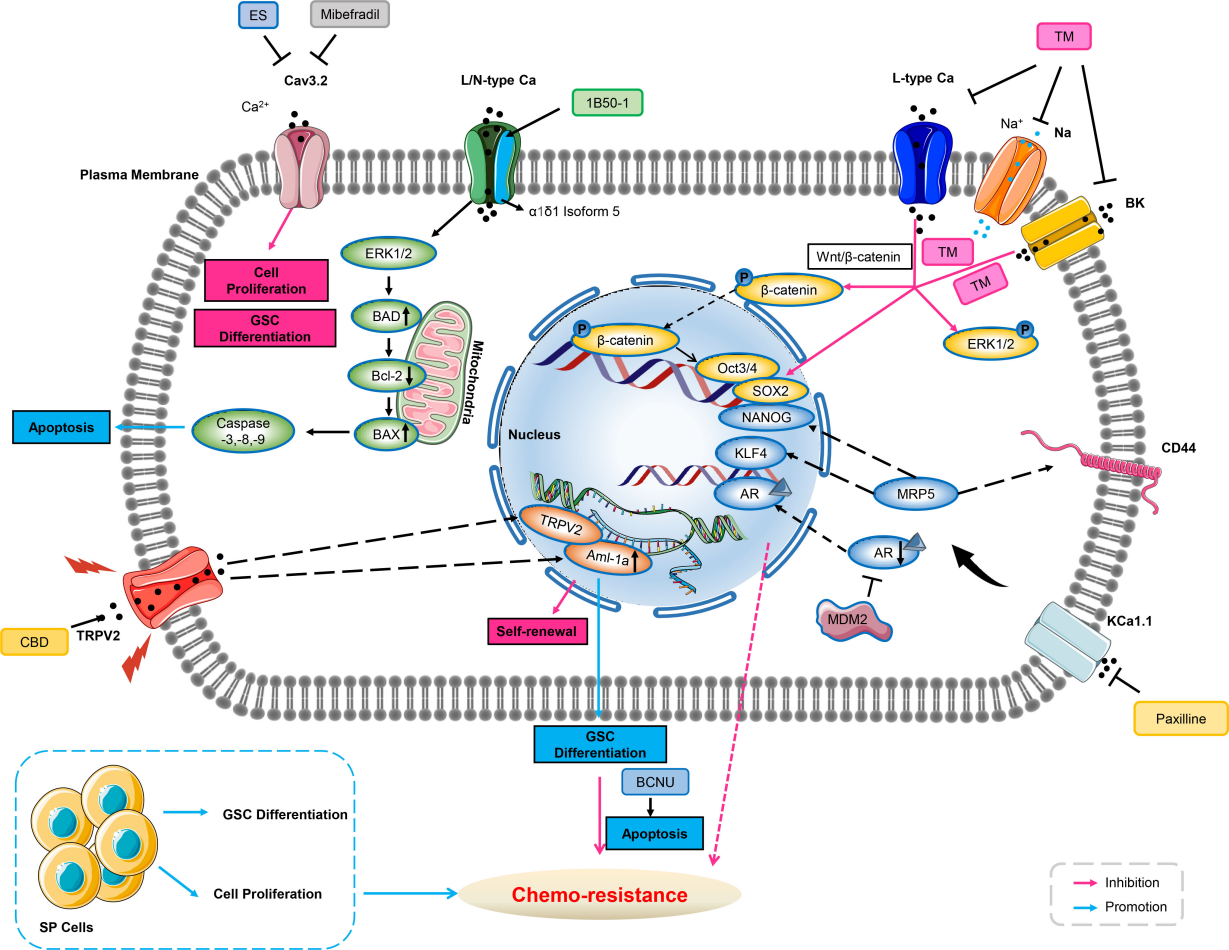
Ion channels involve in chemo-resistance through cancer stem cell. Blockade of Cav3.2 by either mibefradil or ES inhibits cell proliferation and GSC differentiation. 1B50-1 positive HCCs exhibits tumor-initiating cells property, treatment of the monoclonal antibody of 1B50-1 activates ERK1/2 signaling pathway to result in apoptosis of TICs, thus overcome chemo-resistance. Voltage-gated calcium channels, sodium channels, or BK channels are over-expressed in ovarian CSCs. TM inhibits these ion channels’ currents, and reduces transcription factors of Oct3/4 and SOX2 *via* β-catenin signaling and ERK1/2 phosphorylation related to stemness and cell growth of ovarian CSCs, therefore abating chemo-resistance. Inhibition of KCa1.1 by its selective blocker of Paxilline can overcome antiandrogen acquired resistance due to MDM2 in human prostate cancer stem cells, meanwhile, this inhibition also overcomes acquired resistance of DOX due to MRP5 in LNCaP stem cells characterized with stemness markers of NANOG, CD44 and KLF4. CBD increases expression of AML-1a which subsequently binds to TRPV2 promoter to enhance transcription, and then induces GSCs differentiation, thus increasing the sensitivity to BCNU by triggering GSCs apoptosis. GSC, glioblastoma stem-like cells; ES, endostatin; Bcl-2, B-cell lymphoma-2; BAD, Bcl-2 associated death promoter; HCC, hepatocellular carcinoma; TICs, tumor-initiating cells; ERK1/2, extracellular regulated protein kinases1/2; DOX, doxorubicin; MRP, multidrug-associated protein; AR, androgen receptors; CBD, cannabidiol; AML, Acute myeloid leukemia; GSC, glioblastoma stem-like cells; BCNU, carmustine; MDM2, murine double minute 2.

Since increased oxidative stress may lead to oxidative damage of cellular components and result in cell death, cancer cells surviving from endogenous stress and developing into tumors must evolve exquisite mechanisms for adaption of ROS stress. This adaptation can reduce the sensitivity of cancer cells to chemo-therapy or even develop drug resistance (128). TRPV2 has been found up-regulated in human hepatocellular carcinoma cells. And it could significantly enhance the cytotoxicity of H_2_O_2_-mediated oxidative stress, suggesting up-regulated TRPV2 attenuated oxidative adaptation in hepatocellular carcinoma cells. Over-expression of TRPV2 in H_2_O_2_-treated hepatocellular carcinoma cells exacerbated the inhibition of AKT and Nrf2, whereas the activation of p38 and JNK has been enhanced at the early stage of cell death. Interestingly, the increased expression of TRPV2 in HepG2 cells enhanced the effect of stress-related chemicals to induce cell death. The results suggested that TRPV2 was an important enhancer of H_2_O_2_-induced cytotoxicity. These findings suggested that the inhibition of oxidative adaptation may abate drug resistance (129) (**Figure 2**).

## Ion channels modulate chemo-resistance through cancer cell metabolism

Abnormal cell metabolism was an important hallmark of cancer (130). Intracellular metabolism of glucose, amino acids and lipids support cancer cell growth, metastasis and survival. In addition, abnormal cellular metabolism contributed to the acquisition of cancer stem cells (131, 132). Usually cancer cells increase glucose uptake 10 times more than normal cells, and convert glucose to lactate even in the presence of oxygen (133). Glycolysis, the central pathway of glucose metabolism, has been shown to maintain cancer stemness and induce chemo-resistance (134, 135).

### Transient receptor potential ion channels

**Figure 3:**
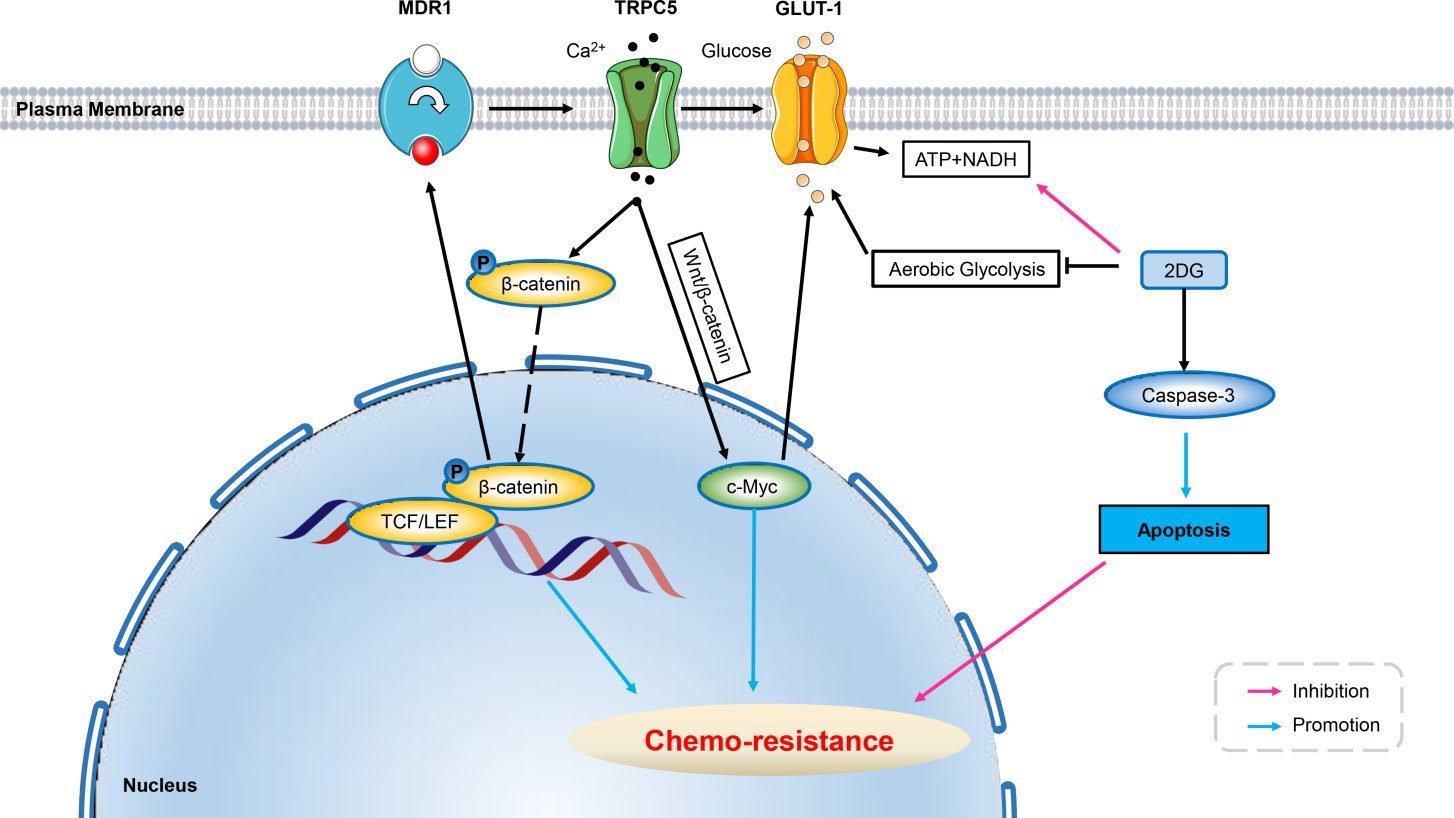
Ion channels develop resistance to chemo-therapy involving cell metabolism. Over-expression of TRPC5 leads to increased intracellular Ca^2+^ which facilitates the translocation of β-catenin into the nucleus, and induces ABCB1 (MDR1) expression, potentiating resistance to 5-Fu in HCT-8/5-Fu cells. Up-regulated TRPC5 in human colorectal cancer cells activates the Wnt/β-catenin signaling pathway, which has been shown to induce GLUT1 expression through its target gene c-Myc, thus exacerbating chemo-resistance. Using glycolysis inhibitor of 2DG can significantly increase the level of cleaved Caspase-3 in HCT-8/5-FU cells, thereby inducing apoptosis and reverse chemo-resistance. ABCB1, ATP-binding cassette, subfamily B, member 1; GLUT, glucose transporter; 5-Fu, 5-Fluorouracil; 3-BP, 3-bromopyruvate; 2DG, 2-Deoxy-D-glucose; Wnt/β-catenin, Canonical Wnt/β-catenin pathway.

Studies have found that up-regulation of TRPC5 expression is associated with chemo-resistance in human colorectal and breast cancers by altering Ca^2+^ influx (136, 137). Numerous studies have shown that up-regulation of TRP channels play inversely roles in cancer, ranging from induction of apoptosis to facilitation of survival (138). The efflux of intracellular calcium is an ATP-dependent process. In non-malignant cells, oxidative phosphorylation was the main source of ATP under physiological conditions, and inhibition of mitochondrial metabolism disrupted intracellular calcium homeostasis and leads to cell death (139, 140). Aerobic glycolysis plays an important role in tumor progression, metastasis and recurrence by providing ATP and metabolites (130, 141). In addition, ATP produced by aerobic glycolysis was recently shown to play a key role in intracellular calcium efflux and homeostasis in malignant tumor cells (142). Several studies have found elevated aerobic glycolysis in drug-resistant cancer cells, which was essential for maintaining chemo-resistance (143–146). It is believed that glycolysis-derived ATP is crucial for drug-resistant cancer cells to cope with sustained chemo-therapeutic stress (143, 147), including enhanced drug inactivation, mutation of survival-related genes, deregulation of growth factor signaling pathways, increased expression of anti-apoptotic genes and/or activation of intracellular survival signals (148). Inhibition of glycolysis caused a significant decrease in ATP production, increased [Ca^2+^]i levels, caspase-3 cleavage, and increased apoptosis in colorectal cancer resistant cells, reversing resistance to chemo-theraputic agent (Fluorouracil, 5-FU). Since [Ca^2+^]i efflux is ATP-dependent and elevated [Ca^2+^]i levels have been shown to trigger apoptosis (149–151), a plausible explanation for the increased expression of cleaved caspase-3 after glycolysis inhibition was the deprivation of glycolysis-derived ATP, which subsequently leading to elevated [Ca^2+^]i levels. Furthermore, BAPTA-AM (a calcium chelator) reduced 2DG (glycolysis inhibitor) -induced increase in caspase-3 cleavage and apoptosis rate. This suggested an important role of increased glycolysis in TRPC5-induced chemo-resistance by supporting ATP to maintain the dynamic balance of intracellular [Ca^2+^]i. Further studies in patients with advanced colorectal cancer implementing chemo-therapy showed that the effect of high TRPC5 expression on chemo-resistance was dependent on high GLUT1 expression. The up-regulated expression of TRPC5 was shown to activate glycolysis in human colorectal cancer cells *via* the Wnt/β-catenin signaling pathway which has been shown to induce GLUT1 expression through c-Myc (136, 152). Moreover, TRPC5 activates Wnt/β-catenin, which promotes glycolysis by mediating Ca^2+^ influx, providing ATP and preventing [Ca^2+^]i overload, thereby inducing chemo-resistance. Therefore, high “TRPC5 glycolysis” is closer to chemo-resistance than high TRPC5 (153) (**Figure 3**).

## Conclusions and outlook

Chemo-resistance is a major impediment to cancer therapy which easily leads to cancer recurrence. Resistance is a complex phenomenon involving multiple mechanisms, including activation of signaling pathways, improved anti-apoptotic capacity, and increased extrusion of therapeutic compounds. Despite many drugs/therapies are currently available in oncology, resistance to therapy hinders complete success of treatment and leads to mid- to long-term recurrence. Ion fluxes have been reported to modulate the response of cancer cells to several chemo-therapeutic agents (5). Moreover, the regulation of apoptosis by ion channels is well described, so it would be interesting to understand their significance in therapeutic-resistant compound.

Notably, Ion channels can be modulated by a wealth of natural compounds and small molecules. Currently, many classical drugs targeted ion channels for treatments are already on the market and even on the essential medicine list of WHO. It has been suggested that repurposing these marketed drugs for the treatment of cancer may be a practical option, especially for the refractory cancer with no drugs available (154). And such attempts may provide a new avenue to tackle chemo-resistance in cancer therapy.

Of course, when targeting ion channels for chemo-therapeutic drug development, cardiac safety and risk mitigation should be considered due to regulatory requirements. It is well-established that the cardiac K^+^, Na^+^, and L-type Ca^2+^ channels are associated with long QT syndrome (LQTs). Specifically, mutations in the α subunit of hERG channels initiate genetic LQTs and inhibition of hERG channel could lead to cardiac arrhythmia. Accordingly, strategies should be employed when screening new drug candidates targeted hERG channels. On the one hand, high-throughput assays can be used to screen safe hERG inhibitor without obvious cardiovascular toxicities (155). On the other hand, specifically targeting hERG in cancer cells without affecting hERG in healthy tissues could overcome the critical difficulties (156). More pertinently, study revealed that blockade of hERG increased the substrate for arrhythmogenesis, and concurrently inhibiting L-type Ca^2+^ channels reduced arrhythmogenic substrate and EADs (Ca^2+^-induced early after depolarizations) which could initiate cardiac arrhythmia. This contradict effects of both contribution to arrhythmogenesis and to its amelioration suggest that besides assessment on the particularly important hERG channel, the safety assays should concern of screening the effects of drugs on other cardiac ion channels including Na^+^ and L-type Ca^2+^ channels (157–159).

The review reported herein describes the regulatory role of ion channels in chemo-resistance *via* different mechanisms of tumor microenvironment, tumor stem cells, and tumor cell metabolism (**Table 1**). Although many studies have reported the relationship between chemo-resistance and ion channels, relatively few studies have provided the complete mechanism. Therefore, it seems necessary to update the understanding of the mechanism involving ion channels in order to enable potential therapeutic associations, including ion channel modulators, to ideally overcome resistance to chemo-therapeutic compounds. For several decades, the pharmaceutical industry has successfully developed ion channel blockers for the treatment of cardiac or psychiatric disorders. However, their therapeutic efficacy has not been extensively studied in clinical treatment for cancer. Studies demonstrated that calcium channel blocker of verapamil significantly improved survival in patients with anthracycline-resistant metastatic breast cancer when used in combination with chemo-therapy (160). In addition, the T-type calcium channel blocker mibefradil hydrochloride acts as a radio-sensitizer by enhancing the effects of hypofractionated radiation in patients with recurrent glioblastoma. The sodium channel blocker of Riluzole has been described for the treatment of cancer in patients with melanoma brain metastasis (161, 162). These ion channel modulators are all repurposed as chemo-therapy drugs. Hopefully, accumulating data on chemo-resistance conferred by ion channels will help repurpose ion channel modulators in clinical trials to improve cancer treatment (6).

**Table 1:** Ion Channels Modulate in Cancer Chemo-resistance.

Channel	Expression	Modulation in Chemo-resistance	Biological Roles	Signaling Pathways	References
Cav3.2 (↓)	GBM	Tumor microenvironment	Cell proliferation (-)	AKT/mTOR	(73)
			Apoptosis (+)	BAX/Caspase-9/PARP	(73)
			Chemo-resistance (-)		(73)
		Cancer stem cell	Cell proliferation (-)	n.d.	(73, 112–114)
			GSC differentiation (-)		(73, 112–114)
KCa1.1 (↑)	GBM	Tumor microenvironment	Cell migration (+)	n.d.	(76)
			Chemo-resistance (+)		(76)
KCa1.1 (↓)	PC	Cancer stem cell	Chemo-resistance (-)	MRP5/MDM2/AR	(123)
	OC	Cancer stem cell	CSC growth (-)	Wnt/β-catenin	(124)
TRPC6 (↑)	HCC	Tumor microenvironment	EMT (+)	STAT3/AKT/ERK1/2	(77)
			Chemo-resistance (+)		(77)
TRPM8 (↑)	PC	Tumor microenvironment	Fibronectin adhesion (+)	n.d.	(79)
			Cell proliferation (+)		(79)
			Invasion (+)		(79)
TRPM7 (↑)	PC	Tumor microenvironment	Cell migration (-)	RACK1/HSP90	(80)
			Invasion (-)		(80)
			Chemo-resistance (-)		(80)
VGCC α1δ1 (↓)	HCC	Cancer stem cell	Self-renewal (-)	ERK1/2/Caspase-3, -8, -9	(120)
			Tumor formation capacities (-)		(120)
			Apoptosis (+)		(120)
VGCC α1δ1 (↑)	HCC	Cancer stem cell	Self-renewal (+)	CXCL11/ERK1/2	(122)
			Cell proliferation (+)		(122)
			Chemo-resistance (+)		(122)
L-type VGCC (↓)	OC	Cancer stem cell	CSC growth (-)	Wnt/β-catenin	(124)
TRPV2 (↑)	GBM	Cancer stem cell	CSC growth (-)	AML-1/PI3K/AKT	(127)
			GSC differentiation (+)		(127)
			Autophagy (+)		(127)
			GSC proliferation (-)		(127)
			Self-renewal (-)		(127)
			Apoptosis (+)		(127)
			Chemo-resistance (-)		(127)
	HCC	Cancer stem cell	Cell growth (-)	AKT/p38/JNK1	(129)
			Cell death (+)		(129)
TRPC5 (↓)	CRC	Cancer cell metabolism	Chemo-resistance (-)	Wnt/β-catenin	(136)
TRPC5 (↑)	CRC	Cancer cell metabolism	Chemo-resistance (+)	GLUT1/c-Myc	(152)

↑, activation; ↓, inhibition; +, increase; -, decrease. AKT, protein kinase B; mTOR, mammalian target of rapamycin; ERK1/2, extracellular regulated protein kinases1/2; STAT3, signal transducer and activator of transcription 3; PARP, poly ADP-ribose polymerase; MDM2, murine double minute 2; AR, androgen receptors; MRP, multidrug-associated protein; RACK1, Receptor of activated protein kinase C1; HSP, Heat Shock Protein; CXCL11, Chemokine Ligand 11; Wnt/β-catenin, Canonical Wnt/β-catenin pathway; AML, Acute myeloid leukemia; JNK, c-Jun N-terminal kinase; GLUT, glucose transporter; Glioblastoma (GBM); Prostate cancer (PC); Ovarian Cancer (OC); Hepatocellular carcinoma (HCC); Colorectal Cancer (CRC); n.d., not determined.

## Author contributions

WC designed the research and revised the manuscript. JZ, ML and JX conducted the literature collection. JZ wrote the original draft of the manuscript. All authors contributed to the article and approved the submitted version.

## Funding

This work has been supported by scientific research fund of educational department of Liaoning Province (LZ2020019 to WC).

## Conflict of interest

The authors declare that the research was conducted in the absence of any commercial or financial relationships that could be construed as a potential conflict of interest.

## Publisher’s note

All claims expressed in this article are solely those of the authors and do not necessarily represent those of their affiliated organizations, or those of the publisher, the editors and the reviewers. Any product that may be evaluated in this article, or claim that may be made by its manufacturer, is not guaranteed or endorsed by the publisher.
